# Altered thymocyte development observed in EphA4-deficient mice courses with changes in both thymic epithelial and extracellular matrix organization

**DOI:** 10.1007/s00018-022-04610-w

**Published:** 2022-11-05

**Authors:** Javier García-Ceca, Sara Montero-Herradón, Ana González, Rosa Plaza, Agustín G. Zapata

**Affiliations:** 1 Department of Cell Biology, Faculty of Biology, Complutense University of Madrid, 28040 Madrid, Spain; 2 Health Research Institute, Hospital 12 de Octubre (imas12), 28041 Madrid, Spain

**Keywords:** Thymus, Eph tyrosine kinase receptors, Thymic microenvironments, Cell migration

## Abstract

Eph receptors and their ligands, Ephrins, are involved in the thymocyte-thymic epithelial cell (TEC) interactions, key for the functional maturation of both thymocytes and thymic epithelium. Several years ago, we reported that the lack of EphA4, a Eph of the subfamily A, coursed with reduced proportions of double positive (DP) thymocytes apparently due to an altered thymic epithelial stroma [Munoz et al. in J Immunol 177:804–813, 2006]. In the present study, we reevaluate the lymphoid, epithelial, and extracellular matrix (ECM) phenotype of EphA4^−/−^ mice grouped into three categories with respect to their proportions of DP thymocytes. Our results demonstrate a profound hypocellularity, specific alterations of T cell differentiation that affected not only DP thymocytes, but also double negative and single positive T cell subsets, as well as the proportions of positively and negatively selected thymocytes. In correlation, thymic histological organization changed markedly, especially in the cortex, as well as the proportions of both Ly51^+^UEA-1^−^ cortical TECs and Ly51^−^UEA-1^+^ medullary TECs. The alterations observed in the expression of ECM components (Fibronectin, Laminin, Collagen IV), integrin receptors (VLA-4, VLA-6), chemokines (CXCL12, CCL25, CCL21) and their receptors (CXCR4, CCR7, CCR9) and in vitro transwell assays on the capacity of migration of WT and mutant thymocytes suggest that the lack of EphA4 alters T-cell differentiation by presumably affecting cell adhesion between TECs and T-TEC interactions rather than by thymocyte migration.

**Supplementary Information:**

The online version contains supplementary material available at 10.1007/s00018-022-04610-w.

## Introduction

The thymus is a primary lymphoid organ where lymphoid progenitor cells phenotypically and functionally mature with the concourse of the thymic stroma. Previously, we demonstrated that Eph, a family of protein tyrosine kinases, and their ligands, Ephrins, were expressed on the surface of both thymic epithelial cells (TECs) and thymocytes (T) [1, 2]. We also showed that some of these, EphB2 and EphB3, and their ligands Ephrins B1 and B2, governed the thymic epithelial histogenesis, and controlled T cell differentiation by mediating T-TEC interactions [3, 4]. In other systems, Eph and Ephrins control morphogenesis, cell positioning and cell migration [5–7]. Regarding the Eph family A, we first reported its expression in rat thymus [1] and later in the phenotype of EphA4-deficient thymuses [8]. In these studies, the absence of EphA4 resulted in thymic hypocellularity and decreased proportions of DP (CD4^+^CD8^+^) thymocytes in a process apparently regulated by the thymic stroma, principally the thymic epithelium, which would impede the proper journey of developing thymocytes throughout the distinct thymic epithelial compartments, and also their immune education [8].

However, these results required further confirmation because the thymic phenotype of EphA4-deficient mice was variable due to the outbred condition of the studied CD1 mouse strain. Thus, most EphA4^−/−^ mice did not show low percentages of DP thymocytes although they exhibited other alterations in thymocyte maturation, and the condition of mutant thymic epithelium needs further evaluation [8]. On the other hand, EphA4 has been reported to modulate TCR-CD3 complex signaling [9], chemokine-dependent cellular migration and integrin-mediated cell adhesion [10], all key processes for a proper functioning of the thymus.

Accordingly, we have reevaluated the condition of EphA4-deficient thymuses by comparing three groups of mutant mice: the first with high proportions of DP thymocytes, a second intermediate group of mutant thymuses exhibiting about 50–70% of DP cells, and a third group with a low proportion (about 5–30%) of DP thymocytes. Apart from the phenotypical characterization of distinct T cell subsets defined by CD4/CD8 expression, we pay special attention to positive and negative selection and to the percentage of Treg cells, as well as to the histological organization of the thymic cortex and medulla, their cellular composition and the condition of molecules, such as chemokines and extracellular matrix (ECM) components (Fibronectin -FN-, Laminin -LN- and collagen IV -Col IV-) and their receptors involved in the migration of thymocytes throughout the thymic epithelial network.

As reported previously, a collapse in the thymic cortex occurs in mutant thymuses which our current results correlate with the gradual decrease in DP cell proportions, whereas the medullary network appeared extended. Remarkably, this histological organization courses with increased proportions of Ly51^+^UEA-1^−^ cortical (c) TECs and reduced percentage of Ly51^−^UEA-1^+^ medullary (m) TECs, presumably by defective maturation of MHCII^+/lo^CD80^−^ immature mTECs. In addition, changes in the ECM components and their receptors seem to affect cell adhesion rather than thymocyte migration, whereas chemokine/chemokine receptor interactions seem to be less relevant for the phenotypes found. Accordingly, we propose a model to explain the phenotype of different EphA4-deficient thymuses in which the lack of EphA4 signaling results in altered cell adhesion of TECs and T-TEC interactions that presumably affect the functional education of developing thymocytes.

## Methods

### Animals

EphA4-deficient mice (*Mus musculus, L*.) were originally provided by Dr. P. Charnay (l’Institut National de la Sante et de la Recherche Medicale, Ecole Normale Superievre, Paris, France) and the mutation was transferred in a CD1 background. Mutant mice (EphA4^−/−^) from heterozygous (EphA4^+/−^) parents were analyzed and compared with littermates [EphA4^+/+^, Wild Type (WT)] at 3 weeks-old. Animals were maintained in pathogen-free conditions in the animal facilities of the Complutense University of Madrid (UCM). Experimental procedures were developed in accordance with the regulations of the University Ethic Committee for Animal Research and the Regional Government of Madrid.

### Cell suspensions

For thymocyte and TEC suspensions, thymuses were cleaned from connective tissue, coagulated blood, or adipose tissue. Thymocyte suspensions were mechanically obtained with RPMI 1640 (RPMI) medium supplemented with 2% fetal bovine serum (FBS). Cell suspensions were filtered, centrifuged at 1500 rpm for 5  minutes (min.) at 4 ºC and the pellet resuspended in RPMI medium with 2% FBS. Enriched TEC suspensions were obtained by cutting clean thymic lobes, transferred into a Falcon tube containing cold 1 × phosphate buffer saline (PBS) and resuspended twice to promote the release of the contained thymocytes. Supernatants were removed in each step and the final fragments were enzymatically disrupted using Liberase TM (Roche) at 1 U/mL together with DNAse I at 0.1 mg/mL (Roche) for 20 min. at 37 ºC. Fragments were completely disaggregated mechanically, and the cell suspension filtered and washed in FACS buffer (PBS with 2% FBS and 10 mM EDTA), centrifuging at 1500 rpm for 5 min. at 4 ºC. Finally, the pellet was resuspended in FACS buffer. In all cases, cell counting was performed in a Neubauer-type haemocytometer discarding the dead cells.

### Flow cytometry

A total of 0.3 × 10^6^ or 1 × 10^6^ cells were used to identify superficial or intracellular thymocyte cell markers, respectively, and 1–2 × 10^6^ cells were used for TEC characterization. Cell suspensions were stained with specific antibodies (Online Resource 1) for 15 min. at 4 ºC in darkness except anti-CCR7 and its control isotype that were incubated for 30 min. at 37 ºC. After incubation, samples were washed with PBS and centrifuged at 1500 rpm for 5 min. at 4 °C. The detection of UEA1-biotin was performed by incubating the samples again with streptavidin (Online Resource 1) for 15 min. at 4 °C and subsequently washed. For detection of Caspase-3 and FoxP3 intracellular markers, after superficial antigen staining, cell suspensions were fixed and permeabilized overnight at 4 ºC with CytoFix/CytoPerm solution (BD Biosciences). Then, samples were washed with PermWash solution (BD Biosciences) at 1500 rpm for 5 min. at 4 ºC. Pellets resuspended in PermWash containing anti-cleaved Caspase-3 or anti-FoxP3 antibodies were incubated for 30 min. at room temperature or 4 ºC, respectively. Finally, samples were washed in PermWash, resuspended in PBS and analyzed in a flow cytometer FACS Calibur or FACS Aria III (BD Biosciences) from the Cytometry and Fluorescence Microscopy Centre of the UCM. Dead cells were excluded from the analysis according to size (FSC) and complexity (SSC) parameters. All data obtained were analyzed with the software FCS Express III (De Novo Software). A minimum of 7 (WT), 6 (DP^hi^), 3 (DP^int^), 3 (DP^low^) mice were used for thymocytes subset analysis or 5 (WT), 5 (DP^hi^), 4 (DP^int^), 3 (DP^low^) for TEC analysis.

### Apoptosis and cell cycle analysis

For apoptosis analysis, thymic cell suspensions or suspensions enriched in TECs were washed in Annexin buffer (BD Biosciences) and incubated with AnnexinV (Online Resource 1) plus anti-CD4, anti-CD8, anti-TCRβ or anti-EpCAM, anti-CD45, anti-Ly51 and UEA1-biotin for 20 min. at room temperature in the dark. Detection of UEA1-biotin was performed using a secondary antibody and then resuspended in Annexin buffer. Before analysis, cell suspensions were stained with Sytox Blue Dead Cell Stain (ThermoFisher Scientific). Apoptotic cells were defined as AnnexinV^+^/SytoxBlue^−^ cells. For cell cycle, cells were washed in PBS and incubated in Cytofix/Cytoperm solution (BD Biosciences) at 4 ºC for 30 min. in the dark. After that, cells were washed twice using PermWash buffer solution and incubated in PermWash solution containing 5 μg/mL of Hoechst 33342 (ThermoFisher Scientific) at 4 ºC for 1 hour (h) and 30 min. in the dark. Cycling cells were defined as cells in S + G_2_/M cell cycle phase. In both cases, cells were analyzed in a FACS AriaIII cytometer (BD Biosciences) at the Cytometry and Fluorescence Microscopy Centre of the UCM. A minimum of 7 (WT), 6 (DP^hi^), 3 (DP^int^), 3 (DP^low^) mice were used for thymocyte or 6 (WT), 5 (DP^hi^), 4 (DP^int^), 3 (DP^low^) for TEC analysis.

### Immunofluorescence

Isolated thymuses were embedded in Tissue-Tek OCT Compound (Sakura) and frozen in liquid nitrogen. 8 μm-thick cryosections were fixed in acetone at room temperature for 10 min., air dried and stained with different primary antibodies (Online Resource 2) during 1 h at room temperature or for 2 h for anti-CCL21, anti-CCL25 and anti-CXCL12. After washing in cold PBS 1 × three times for 5 min., they were detected using specific secondary antibodies conjugated with different fluorochromes (Online Resource 2) incubated in the dark during 45 min. at room temperature. Sections were then washed and mounted with antifade Prolong Gold (Molecular Probes), observed in a Zeiss Axioplan 2 microscope, photographed with a Spot 2 digital camera, and analyzed using Metamorph software (MDS Inc.) at the Cytometry and Fluorescence Microscopy Centre of the UCM.

### Immunohistochemistry semiquantitative analysis

For the semiquantitative analysis of the expression of LN, FN and Col IV, as well as CCL25, CCL21 and CXCL12, a minimum of 10 non-overlapping histological sections from three different thymuses were analyzed. The relative expression of the different molecules was obtained by calculating the area in pixels^2^ for each of them with respect to the total cortical or medullary area (pixels^2^) in each histological section × 100. The number of AIRE^+^ cells was determined by counting the number of positive cells in the K5^+^ medullary area (pixels^2^) and referred to as AIRE^+^/K5^+^. All semi-quantitative analysis was carried out using Adobe Photoshop CS6 software (Adobe Systems Inc.).

### Cell migration assay

For in vitro migration assays, 1 × 10^6^ total thymocytes were seeded on 24-well transwell plates containing nitrocellulose inserts with 5 μm pore size (Corning Costar). Previously, the inserts were coated with BSA (Sigma-Aldrich) or LN (Corning) (10 μg/ml) for 1 h at 37 ºC and subsequently washed twice with sterile PBS. Next, 600 μl of RPMI 1% BSA or medium supplemented with chemokines CCL21 or CCL25 (100 ng/ml) (Biolegend) were added to the wells. 100 μl of total thymocyte suspensions were added to the insert and incubated for 18 h at 37 ºC and 5% CO_2_. Finally, the button volume of the well was collected, centrifuged for 5 min. at 1500 rpm and the pellet resuspended in PBS. Cell numbers were determined in a haemocytometer and the proportion of the different subpopulations of migrating thymocytes was obtained by staining the cell suspension with anti-CD4 and anti-CD8 antibodies and analyzed by flow cytometry. The percentage of migrating cells of each cell subpopulation was calculated by dividing the number of recovered cells by the initial number of seeded cells in the inserts × 100. Three mice for WT, DP^hi^ and DP^low^ were used.

### Statistical analysis

Data were expressed as mean ± standard deviation (SD) and analyzed using one-way analysis of variance (ANOVA) or two-way ANOVA with GraphPad Prism 8 program (GraphPad Software Inc.). For one-way ANOVA data, were subjected to a normality test (D’Agostino-Pearson test) and homoscedasticity test (Brown-Forsythe test). If the data were normal and homoscedastic, one-way ANOVA was applied; if they were normal, but heteroscedastic, the ANOVA Brown-Forsythe and Welch test were used, and when they were not normal, a nonparametric Kruskal–Wallis test followed by the post hoc Tukey, Tamhane T2 or Dunn’s test were used, respectively. After two-way ANOVA, Tukey’s post hoc test was applied. Statistical significance between control and mutant values was indicated as: *p < 0.05; **p < 0.01; ***p < 0.001.

## Results

### The lymphoid phenotype of EphA4-deficient thymuses

**Fig. 1:**
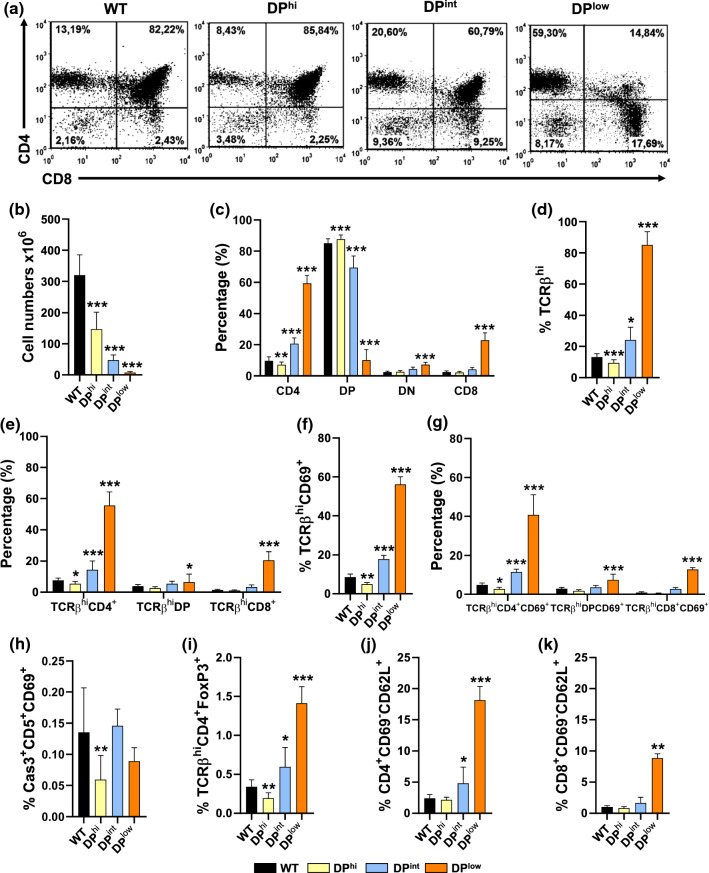
Thymus cellularity and proportions of thymocyte subsets in both WT and EphA4-deficient mice grouped according to their proportions of DP cells. **a** Representative flow cytometry dot plots of both WT (EphA4^+/+^) and EphA4^−/−^ mutant thymuses according to their proportions of DP thymocytes (DP^hi^, DP^int^, DP^low^). **b** Total thymus cellularity. **c** Proportions of different thymocyte subsets according to the CD4/CD8 expression. CD4^+^CD8^−^ SP cells (CD4), DP cells, DN cells, CD4^−^CD8^+^ SP cells (CD8). **d** Percentage of total TCRβ^hi^ thymocytes. **e** Percentage of total mature thymocyte subsets defined by the expression of TCRβ^hi^ and CD4/CD8 markers. **f** Data represent the percentage of total mature positive selected TCRβ^hi^CD69^+^ thymocytes and in different thymocyte subsets (**g**). **h** Percentage of total negative selected Cas3^+^CD5^+^CD69^+^ thymocytes according to the expression of Cleaved caspase-3 (Cas3), CD5 and CD69 cell markers. **i** Frequency of CD4^+^ T regulatory cells according to FoxP3 expression (TCRβ^hi^CD4^+^FoxP3^+^). The proportions of total CD4^+^ (**j**) and CD8^+^ (**k**) CD69^−^CD62L^+^ cells. Data are presented as mean ± standard deviation (SD) and analyzed using the Kruskal–Wallis nonparametric test with Dunn’s post hoc test (**b**, **h**, **j**, **k**), Brown-Forsythe and Welch ANOVA test with Tamhane’s T2 post hoc test (**d**) and one-way (**f**, **i**) or two-way (**c**, **e**, **g**) ANOVA test with Tukey’s post hoc test. *p < 0.05; **p < 0.01; and ***p < 0.001

As indicated, we divided the 3-week-old EphA4-deficient mice into three groups according to the proportions of DP thymocytes found: the first group contained 80–90% of DP thymocytes (DP^hi^ thymuses); a second group exhibited about 50–70% of DP cells (DP^int^ thymuses) and a third group contained only 5–30% of DP thymocytes (DP^low^ thymuses) (Fig. 1a). Remarkably, all mutant mice showed a similar gross-anatomy with a club-foot phenotype in their lower extremities as previously reported [8, 11].

Thymic cellularity was extremely reduced with half the numbers of thymic cells in the mutant mice containing high DP cell proportions and lower numbers in the other ones, in inverse correlation with the proportions of DP thymocytes (× 6 and × 20 reduction in DP^int^ and DP^low^ thymuses, respectively) (Fig. 1b). Apart from changes in the proportions of DP thymocytes, CD4/CD8 expression also showed important differences between the three groups of EphA4-deficient thymuses. In the DP^hi^ mice, the proportions of SP CD4^+^CD8^−^ cells decreased, whereas the values of DN (CD4^−^CD8^−^) and SP CD4^−^CD8^+^ cells remained unchanged (Fig. 1c). The differences were even more evident when EphA4-deficient thymocytes with intermediate or low values of DP cells were compared to those of control, WT mice. In both mutant groups, the decreased proportions of DP cells correlated with significant increases in the proportions of the other T cell subsets, DN and SP (both CD4^+^CD8^−^ and CD4^−^CD8^+^), being more evident in the DP^low^ mice (Fig. 1c).

In correlation with these results, mutant DP^hi^ thymuses showed reduced proportions of total TCRαβ^hi^ thymocytes (Fig. 1d). This was particularly significant in the TCRαβ^hi^CD4^+^ cell subset (Fig. 1e) that seemed to correspond to positively selected TCRαβ^hi^CD69^+^ thymocytes (Fig. 1f) and suggested that increased proportions of DP cells (Fig. 1c) principally corresponded to the most immature TCRαβ^lo/med^ DP T-cell subset. On the contrary, DP^int^ and DP^low^ thymuses exhibited significantly increased proportions of total TCRαβ^hi^ cell populations (Fig. 1d, e), in correlation with significantly greater proportions of positively selected CD69^+^ thymocytes (Fig. 1f) that affected both DP and SP (CD4^+^CD8^−^ and CD4^−^CD8^+^) thymocytes (Fig. 1g). The proportions of total negatively selected Cas3^+^CD5^+^CD69^+^ thymocytes (Fig. 1h), as well as those of FoxP3^+^CD4^+^ Treg cells (Fig. 1i) significantly decreased in the DP^hi^ thymuses. Remarkably, in both DP^int^ and DP^low^ mice, there were no differences in the proportions of negatively selected thymocytes (Fig. 1h), but the proportions of Treg cells significantly increased (Fig. 1i). Finally, SP thymocytes undergo maturation in the medulla, where acquire other markers such as CD62L and S1PR before egress from the thymus [12, 13]. Because increased proportions of SP thymocytes observed in some of the studied mutants could be related to an accumulation of mature thymocytes in the thymic medulla, we analyzed the expression of CD69 and CD62L, defining the CD69^−^CD62L^+^CD4^+^ (Fig. 1j) and CD8^+^ cells (Fig. 1k) as mature thymocytes, whereas DP^hi^ thymuses showed no significant changes in these populations, both DP^int^ and DP^low^ EphA4-deficient thymuses exhibited increased values of these cells, especially in thymuses containing the lowest proportions of DP thymocytes (Fig. 1j, k).

**Fig. 2:**
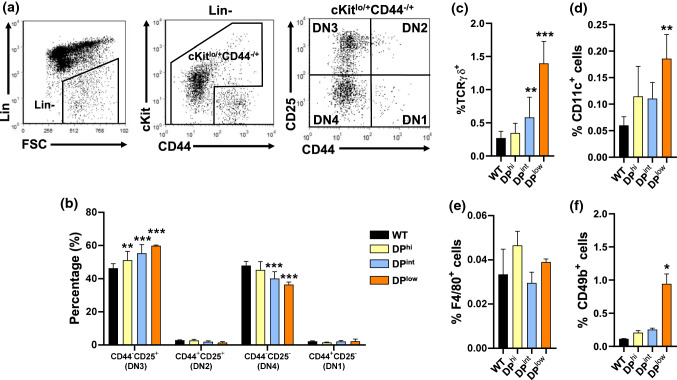
Proportions of different cell subsets included in the DN cell compartment of both WT and EphA4-deficient mice. **a** Flow cytometry strategy to analyze DN1-DN4 thymocyte subsets based on CD44 and CD25 expression gated on Lin^−^ckit^lo/+^CD44^−/+^cells. **b** Percentage of DN1-DN4 thymocyte subsets. Proportions of total TCRγδ^+^ thymocytes (**c**), CD4^−^CD8^−^CD45^+^CD11c^+^ DCs (CD11c^+^) (**d**), CD4^−^CD8^−^CD45^+^F4/80^+^macrophages (F4/80^+^) (**e**) and CD4^−^CD8^−^CD45^+^CD49b^+^ NK cells (CD49b^+^) (**f**). Data are presented as mean ± SD and analyzed using Kruskal–Wallis nonparametric test with Dunn’s post hoc test (**c**, **f**) and one-way (**d**, **e**) or two-way (**b**) ANOVA test with Tukey’s post hoc test. *p < 0.05; **p < 0.01; and ***p < 0.001

In the case of DN thymocytes, it is important to clarify the origin of the variations observed in the different EphA4 mutant thymuses, because this cell subset includes other cell lineages apart from TCRαβ^+^ T lymphocytes [13] and can be subdivided into other subpopulations. In this respect, we first analyzed the changes in T cell subsets (DN1-DN4) defined by CD44/CD25 (DN1 (CD44^+^CD25^−^), DN2 (CD44^+^CD25^+^), DN3 (CD44^−^CD25^+^) and DN4 (CD44^−^CD25^−^) expression within the ckit^lo/+^CD44^−/+^ lineage negative cell compartment (Fig. 2a). In the DP^hi^ thymuses, the total DP thymocyte proportions significantly increased (Fig. 1c) in correlation with those of DN3 cells (Fig. 2b). The behavior of DN cell subsets in both DP^int^ and DP^low^ thymuses was similar, although more evident in DP^low^ ones, exhibiting decreased proportions of DN4 cells and increased percentages of DN3 thymocytes (Fig. 2b). Because, as above mentioned, the DN cell compartment includes other cell lineages apart from αβ T lymphocytes, we analyzed the contribution of other cell types to the changes observed in this thymic cell compartment of EphA4-deficient thymuses. Whereas no significant variations were evident in the DP^hi^ thymuses (Fig. 2c–f), increased proportions of γδ T cells occurred in both DP^int^ and DP^low^ mutant thymuses (Fig. 2c), whereas those of CD4^−^CD8^−^CD45^+^CD11c^+^ dendritic cells (DCs) (Fig. 2d) and CD4^−^CD8^−^CD45^+^CD49b^+^ NK cells increased, but only significantly in DP^low^ thymuses (Fig. 2f).

**Fig. 3:**
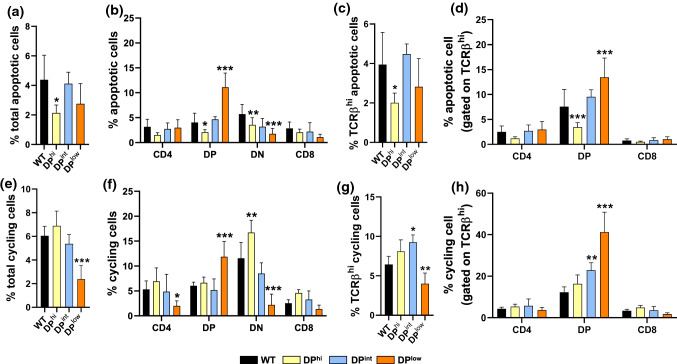
Apoptotic and cycling cells in both WT and EphA4-deficient mice. Apoptosis in total thymocytes (**a**) or in different cell subsets (**b**) on the basis of CD4/CD8 expression. Percentage of apoptosis in total mature TCRβ^hi^ cells (**c**) or in DP and SP thymocytes (**d**). Apoptotic cells were defined as AnnexinV^+^SytoxBlue^−^. Proportions of cycling cells in total thymocytes (**e**), CD4/CD8 cell subsets (**f**), total TCRβ^hi^ cells (**g**) and both DP and SP thymocytes (**h**). Cycling cells were defined as cells in S + G_2_/M cell cycle phase. Data are presented as mean ± SD and analyzed using one-way (**a**, **c**, **e**, **g**) or two-way (**b**, **d**, **f**, **h**) ANOVA test with Tukey’s post hoc test. *p < 0.05; **p < 0.01; and ***p < 0.001

Finally, we evaluated the proportions of both apoptotic (Fig. 3a–d) and cycling cells (Fig. 3e–h) in the total thymic cells and in several T cell subsets. The proportions of total apoptotic thymic cells significantly decreased in DP^hi^ thymocytes (Fig. 3a), in correlation with low values in both DN and DP cell subsets (Fig. 3b), and in the TCRαβ^hi^ DP cells (Fig. 3d). Also, in DP^low^ thymuses, although the proportions of apoptotic cells decreased in the DN cell compartment (Fig. 3b), they significantly increased in both total DP cells (Fig. 3b) and TCRαβ^hi^ DP thymocytes (Fig. 3d).

Likewise, DP^hi^ thymuses of mutant mice exhibited a significant rise in the proportions of cycling thymocytes in the DN cell compartment (Fig. 3f). On the contrary, the total proportion of cycling cells significantly decreased in DP^low^ thymuses (Fig. 3e), reflecting the low cellularity observed in these mutant thymuses (Fig. 1b). Remarkably, although the proportions of cycling DP cells significantly increased in DP^low^ thymuses (Fig. 3f), the percentage of cycling cells decreased in the total TCRαβ^hi^ cell compartment (Fig. 3g) where, however, the proportion of cycling TCRαβ^hi^ DP cells significantly increased (Fig. 3h).

### Thymic histological organization and thymic epithelial cells of EphA4-deficient thymuses

**Fig. 4:**
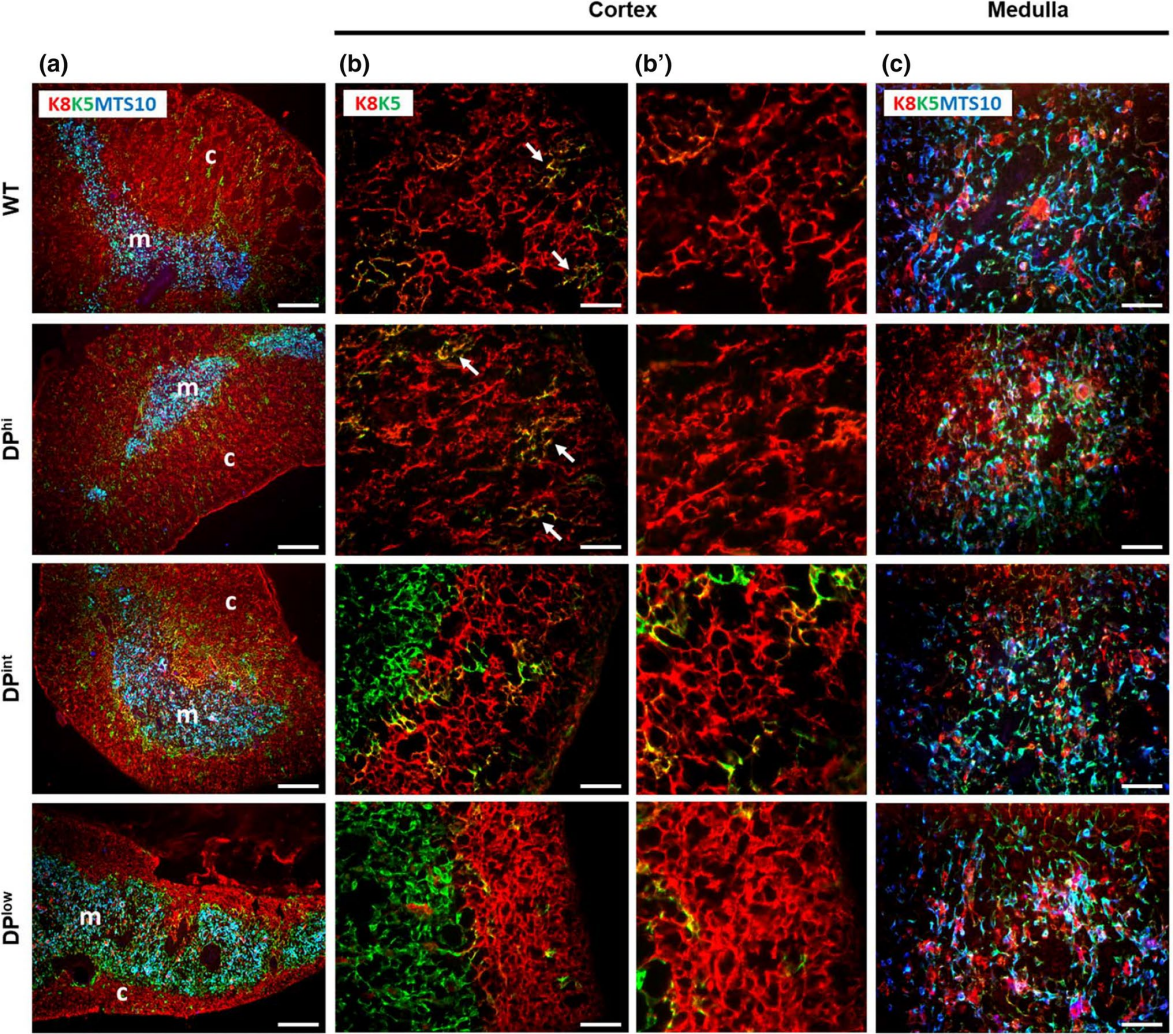
Histological thymus organization of both WT and EphA4-deficient thymuses containing different proportions of DP cells. **a** Figures show a representative organization of thymus parenchyma stained for keratin 8 (K8), 5 (K5) and MTS10 epithelial cell markers. The cortex is mainly formed by K8^+^K5^−^MTS10^−^ cells and the medulla as K8^−^K5^+^MTS10^+^ cells. Arrows indicate immature K8^+^K5^+^MTS10^−^ cells. **b**, **c** Representative thymic epithelial organization at different magnifications of cortex (**b**, **b**’) and medulla (**c**). *c* cortex, *m* medulla. Scale bar, **a** 200 µm, **b** and **c** 50 µm

Because, as mentioned above, previous results [8] have pointed out a key role of the thymic epithelium in the lymphoid phenotype of EphA4-deficient mice, we decided to analyze extensively the thymic histology and possible changes in the proportions of both cTECs and mTECs in the three groups of studied mutants as compared to WT thymuses. The histological organization of DP^hi^ thymuses was quite similar to that of WT ones (Fig. 4), except for increased numbers of immature K8^+^K5^+^MTS10^−^ TECs, which appeared scattered at random throughout the thymic parenchyma (Fig. 4a, b), and a gradual reduction in the cortical area related to the fall in DP cell proportions in both DP^int^ and DP^low^ thymuses (Fig. 1c). In these mutants, the thymic cortex showed flattened, collapsed cTECs (Fig. 4a, b). Gradually, the epithelial cell processes shorten, and the cells become rounded allowing a larger number of cTECs to occupy the spaces left by the thymocytes (Fig. 4b’). Finally, flattened cells constituted several layers parallel to the thymic capsule of collapsed cTECs (Fig. 4b’). On the contrary, the medullary area expanded and the mTECs appeared loosely arranged forming a sponge-like epithelial network (Fig. 4c).

**Fig. 5:**
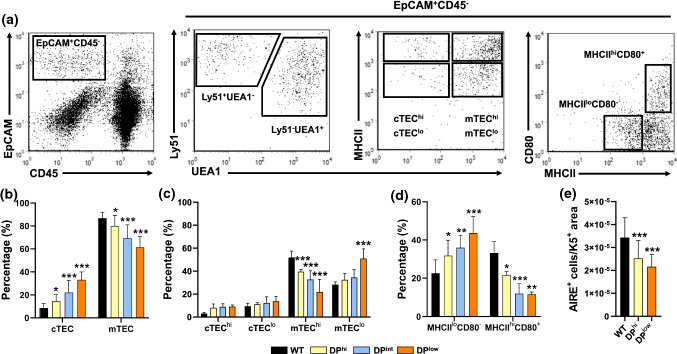
Effects of the lack of EphA4 on the maturation of cortical and medullary TEC subsets. **a** Representative flow cytometry plots showing the gating profile of total EpCAM^+^CD45^−^ TECs and different TEC subpopulations: Ly51^+^UEA-1^−^ (cTEC), Ly51^−^UEA-1^+^ (mTEC), UEA-1^−^MHCII^hi^ (cTEC^hi^), UEA-1^−^MHCII^lo^ (cTEC^lo^), UEA-1^+^MHCII^hi^ (mTEC^hi^), UEA-1^+^MHCII^lo^ (mTEC^lo^), MHCII^lo^CD80^−^ and MHCII^hi^CD80^+^. Proportions of cTEC and mTEC (**b**), cTEC^hi^, cTEC^lo^, mTEC^hi^ and mTEC^lo^ (**c**) as well as MHCII^lo^CD80^−^ and MHCII^hi^CD80^+^ (**d**) cells in control and mutant thymuses. **e** Numbers of AIRE^+^ cells relative to the K5^+^ area of thymic sections of mutant thymuses (DP^hi^ and DP^low^) compared with WT control ones. Data are presented as mean ± SD and analyzed using one-way (**e**) or two-way ANOVA test (**b**, **c**, **d**) with Tukey’s post hoc test. *p < 0.05; **p < 0.01; and ***p < 0.001

Flow cytometry (Fig. 5a) revealed that the proportions of Ly51^+^UEA-1^−^ cTECs in all mutants increased while those of Ly51^−^UEA-1^+^ mTECs significantly reduced (Fig. 5b). In both thymic compartments the changes were inversely proportional to the proportions of DP cells (Fig. 1c). Remarkably, the reduced proportions of mTECs were due to a significant decrease in the more mature UEA-1^+^MHCII^hi^ cells (mTEC^hi^) (Fig. 5c) and MHCII^hi^CD80^+^ mTECs (Fig. 5d), whereas the proportions of immature UEA-1^+^MHCII^lo^ cells (mTEC^lo^) (Fig. 5c) and MHCII^lo^CD80^−^ cells accumulated (Fig. 5d), with significant differences in the DP^low^ thymuses. In the cortical compartment, the proportions of both mature UEA-1^−^MHCII^hi^ (cTEC^hi^) and immature UEA-1^−^MHCII^lo^ (cTEC^lo^) cTECs increased but without significant differences (Fig. 5c). Furthermore, in agreement with the changes observed in the negative selection in EphA4-deficient thymuses (Fig. 1h), in both DP^hi^ and DP^low^ thymuses the proportions of AIRE^+^ cells by medullary area decreased significantly (Fig. 5e).

**Fig. 6:**
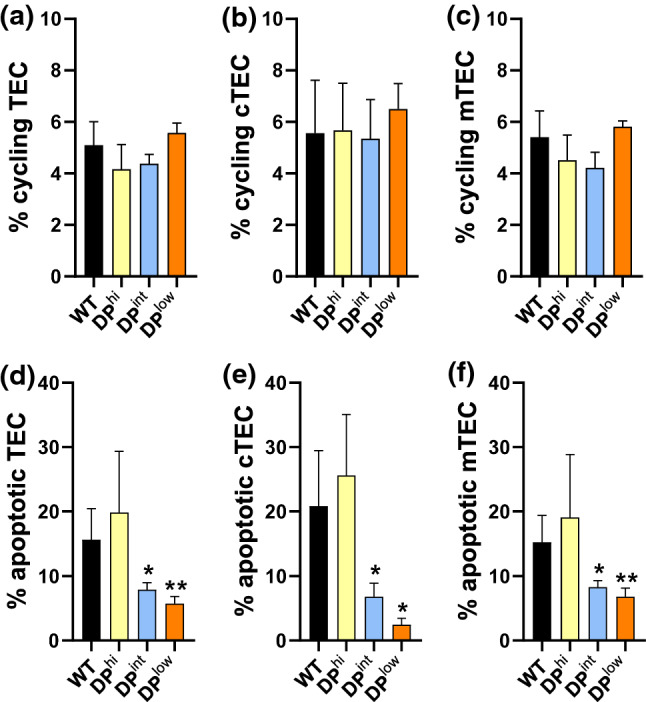
Cell cycle and apoptosis in both WT and EphA4-deficient thymuses. The percentages of cycling (**a**–**c**) and apoptotic (**d**–**f**) cells in total EpCAM^+^CD45^−^ TECs (**a**, **d**), Ly51^+^UEA-1^−^ cTECs (**b**, **e**) and Ly51^−^UEA-1^+^ mTECs (**c**, **f**) in WT and EphA4^−/−^ thymuses were measured by flow cytometry. Apoptotic cells were defined as AnnexinV^+^SytoxBlue^−^ and cycling cells as cells in S + G_2_/M cell cycle phase. Data are presented as mean ± SD and analyzed using Brown-Forsythe and Welch ANOVA test with Tamhane’s T2 post hoc test (**d**, **f**) and one-way ANOVA test with Tukey’s post hoc test (**a**, **b**, **c**, **e**). *p < 0.05; **p < 0.01

On the other hand, the cell cycle of thymic epithelium (Fig. 6a) did not appear to contribute to the reported alterations in the thymic cortex and medulla or, in general, to the low cell content of EphA4-deficient thymuses. Neither did the proportions of cycling cTECs (Fig. 6b) nor those of mTECs (Fig. 6c) underwent significant variations in mutants compared with control WT thymuses. On the contrary, both DP^int^ and DP^low^ TECs did present a significantly reduced apoptosis (Fig. 6d), which affected both cTECs (Fig. 6e) and mTECs (Fig. 6f), whereas DP^hi^ thymuses showed slightly increased proportions of both apoptotic cTECs and mTECs, although the differences were not statistically significant.

### Changes in the extracellular matrix components and expression of their receptors in EphA4-deficient thymuses

**Fig. 7:**
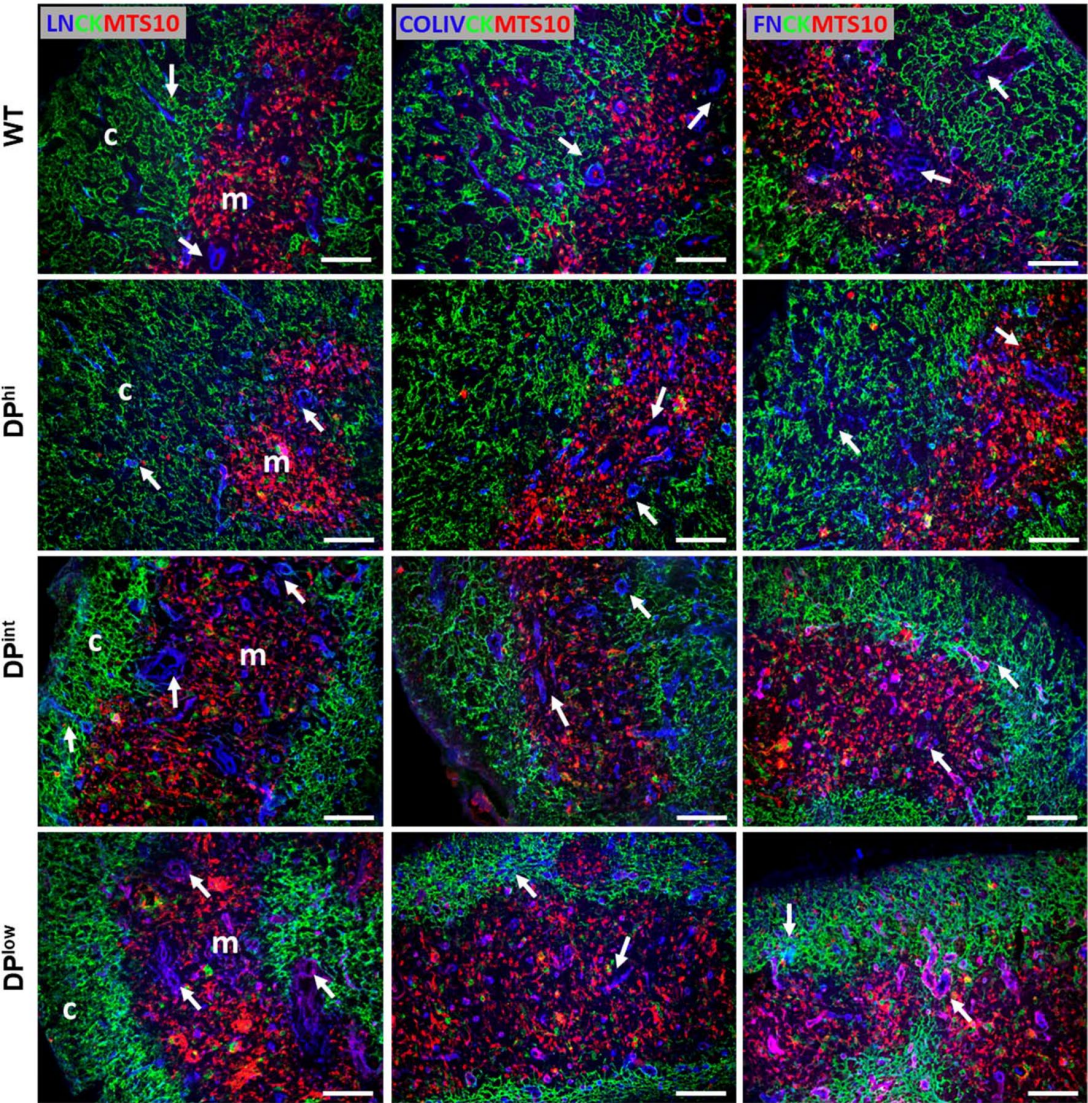
Expression of different ECM components (arrows) in WT and EphA4-deficient thymuses. Expression of laminin (LN), collagen IV (COL IV) and Fibronectin (FN) (blue) in the thymic cortex (c) stained with pan-cytokeratin (CK, green) and in the MTS10^+^ (red) medulla (m) of control and mutant mice. Scale bar: 100 µm

**Fig. 8:**
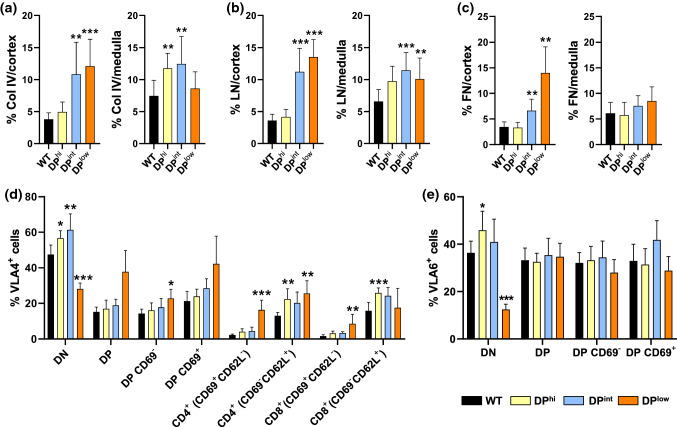
Analysis of ECM components and their receptors in both WT and EphA4^−/−^ thymuses. Semi-quantitative analysis expression of collagen IV (Col IV) (**a**), laminin (LN) (**b**) and fibronectin (FN) (**c**) in the cortex and the medulla from WT and EphA4^−/−^ thymic sections. Flow cytometry analysis of the percentage of VLA4^+^ (**d**) and VLA6^+^ (**e**) cells in different subsets of control and mutant thymocytes. Data are presented as mean ± SD and analyzed using Kruskal–Wallis nonparametric test with Dunn’s post hoc test (**a**–**c**) and one-way ANOVA test with Tukey’s post hoc test (**d**, **e**). *p < 0.05; **p < 0.01; and ***p < 0.001

The expression of FN, LN and Col IV in mutant and WT thymuses (Fig. 7) showed that the most important changes occurred in the ECM of the thymic cortex, principally in both DP^int^ and DP^low^ thymuses. A semiquantitative analysis of the expression of these molecules demonstrated that DP^hi^ thymuses only exhibited a significant increase in Col IV expression in the medulla (Fig. 8a). On the contrary, both DP^int^ and DP^low^ thymuses showed increased expression of LN and Col IV in both cortex and medulla, but FN expression only increased significantly in the thymic cortex (Fig. 8a–c). We then analyzed the expression of VLA-4 and VLA-6 integrin receptors of FN and LN, respectively, in the distinct thymocyte subsets of mutant and WT thymuses (Fig. 8d, e). Remarkably, although in some mutant groups the expression of their specific ligands did not change significantly, the expression of the integrins did. Thus, FN was the ECM component that underwent the least changes in the distinct EphA4-deficient thymuses, but VLA-4^+^ cell proportions showed considerable variations in the three groups of thymuses (Fig. 8d).

As reported previously [14], in WT thymuses VLA-4 is expressed in all T-cell subsets, principally in the DN cells, decreasing in the DP cell compartment and especially in the preselected SP thymocytes (both CD4^+^CD8^−^ and CD4^−^CD8^+^), and increased yet again in the post-selected CD69^−^CD62L^+^ SP cells. In EphA4-deficient mutants, both DP^hi^ and DP^int^ thymuses exhibited profiles of VLA-4 expression quite similar to those observed in WT ones. In DP^hi^ mutants, the proportions of VLA-4 cells increased significantly in the DN cell subset as well as in the post-selected CD4 and CD8 CD69^−^CD62L^+^ thymocytes, whereas in the DP^int^ thymuses, the proportions of VLA-4 cells increased in the same cell subsets, but only significantly in the DN cell compartment (Fig. 8d).

However, a different profile was observed for the presence of VLA-4^+^ thymocytes in the DP^low^ thymuses (Fig. 8d). With the exception of the DN thymocyte stage, where the proportions of VLA-4^+^ cells decreased significantly in the DP^low^ thymuses, the other cell subsets showed increased proportions compared to WT values, with significant differences in both CD4^+^ and CD8^+^CD69^+^CD62L^−^ positively selected cells and CD4^+^CD69^−^CD62L^+^ mature thymocytes (Fig. 8d). On the contrary, except for a significant reduction in the proportion of VLA-6^+^ DN thymocytes in the DP^low^ thymuses as compared to WT ones and increased proportions in DN cells of DP^hi^ thymuses, no variations occurred in the expression of this integrin in the T-cell subsets of EphA4-deficient mice studied (Fig. 8e).

### Expression of chemokines and their receptors in EphA4-deficient thymuses

**Fig. 9:**
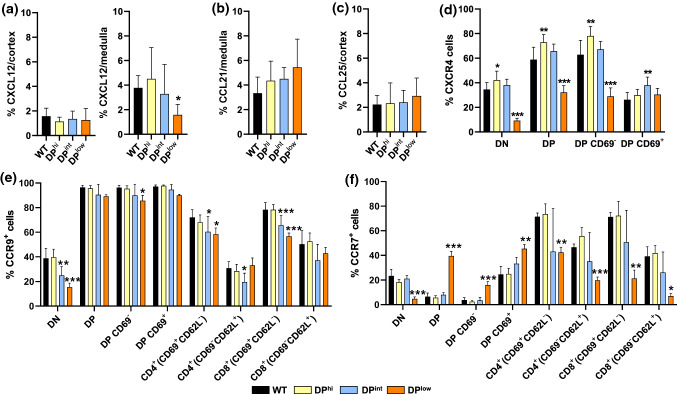
Expression of chemokines and their receptors in both WT and EphA4^−/−^ thymuses. Semi-quantitative analysis of CXCL12 expression in cortex and in medulla (**a**), of CCL21 (**b**) in medulla and CCL25 (**c**) in cortex from WT and EphA4^−/−^ thymic sections. Percentage of CXCR4^+^ (**d**), CCR9^+^ (**e**) and CCR7^+^ (**f**) cells in different subsets of control and mutant thymocytes, evaluated by flow cytometry. Data are presented as mean ± SD and analyzed using one-way ANOVA test with Tukey’s post hoc test. *p < 0.05; **p < 0.01; and ***p < 0.001

We studied the expression profiles of three chemokines, CCL25, CCL21 and CXCL12 (Online Resource 3, Fig. 9a–c) involved in the thymocyte journey throughout the different thymic compartments [14, 15]. Our current results confirmed previous data on the expression of these molecules in the distinct thymic compartments: CCL25 is secreted by cTECs, whereas CCL21 is produced by mTECs and attracts thymocytes from the cortex to the medulla [16]. On the other hand, CXCL12 is expressed by both cortex and medulla and participates in the migration of DP cells throughout the cortex and in their migration to the medulla [14]. However, our semiquantitative analysis did not find changes in the expression of these chemokines in EphA4-deficient mice, except for a significant reduction in the proportions of CXCL12 positive cells in the medulla of DP^low^ mice (Fig. 9a).

The pattern of expression of CXCR4, the CXCL12 receptor, in the thymocyte subsets studied was similar in DP^hi^ and DP^int^ thymuses and opposite to that of DP^low^ thymuses (Fig. 9d). In both DP^hi^ and DP^int^ thymuses, the proportions of DN and DP CXCR4^+^ cells were higher than in WT ones, with significant differences in the case of DP^hi^ mice; however, increased proportions of CXCR4^+^ DP cells correlated with those of CD69^−^ cells in DP^hi^ thymuses and with positively selected CD69^+^ DP cells in DP^int^ thymuses (Fig. 9d). In the case of DP^low^ thymuses, the proportions of CXCR4^+^ cells were significantly lower in DN, total DP and DP CD69^−^ cells, but not in the selected DP CD69^+^ thymocytes (Fig. 9d). The CCR9/CCL25 pair is involved in thymic seeding, DN2/DN3 positioning and the migration of selected thymocytes to the medulla [15]. The CCR9 receptor was principally expressed in both pre- (CD69^−^) and post-selected (CD69^+^) DP thymocytes, decreasing in the SP thymocytes. The proportions of CCR9^+^ cells did not vary in the distinct T cell populations of DP^hi^ thymuses, as compared with WT values (Fig. 9e). On the contrary, both DP^int^ and DP^low^ thymuses showed significantly reduced proportions of CCR9^+^ cells that principally affected the DN cell subset and the positively selected CD4 and CD8 CD69^+^CD62L^−^ thymocytes, but not the CD69^−^CD62L^+^ cell subset (Fig. 9e). CCR7, the receptor of CCL19 and CCL21, is expressed in the earliest stages of T cell development as well as in the SP thymocytes, contributing to their migration from the cortex to the medulla [15]. The general pattern of distribution of CCR7^+^ cells was similar to the CCR9^+^ ones, without changes in the DP^hi^ thymuses and a similar pattern in both DP^int^ and DP^low^, although the differences were only significant in the latter (Fig. 9f). Remarkably, in all the thymuses studied the T cell subsets showed lower values for the proportions of CCR7^+^ cells except for in the DP cell compartment, including both the positively selected CD69^+^ and non-selected CD69^−^ DP cell compartments, which increased significantly (Fig. 9f).

**Fig. 10:**
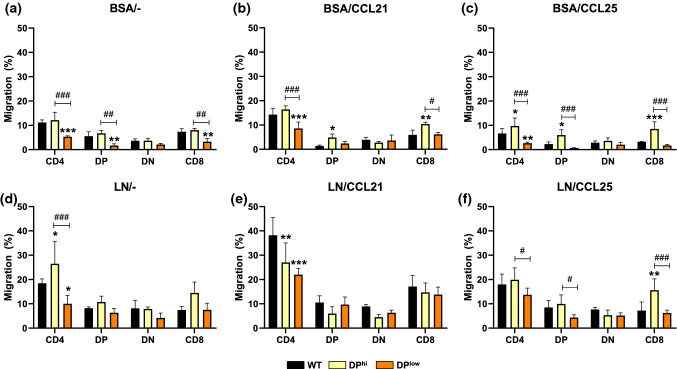
Migration of different thymocyte subsets from WT and EphA4^−/−^ thymuses (DP^hi^ and DP^low^) through BSA or laminin (LN) in the absence (–) or presence of chemotactic stimulus (CCL21 or CCL25). BSA/– (**a**), BSA/CCL21 (**b**), BSA/CCL25 (**c**), LN/– (**d**), LN/CCL21 (**e**), LN/CCL25 (**f**). Data are presented as mean ± SD and analyzed using two-way ANOVA test with Tukey’s post hoc test. Comparison between WT and EphA4 mutants as indicated as *p < 0.05; **p < 0.01; and ***p < 0.001 and between mutant thymuses as ^#^*p* < 0.05; ^##^*p* < 0.01; and ^###^*p* < 0.001

Because developing thymocytes must travel through the thymic parenchyma and, as described, EphA4-deficient thymuses showed important histological changes, we studied the capacities of thymocytes isolated from either DP^hi^ and DP^low^ thymuses to migrate in vitro in transwell in an attempt to correlate presumptive migratory alterations, changes in the thymic epithelium network and the ECM, and defective T cell development. In general, the proportion of migrating thymocytes derived from DP^hi^ thymuses (onward DP^hi^ thymocytes) was higher than that observed with DP^low^ thymocytes, particularly in the case of DP and SP (both CD4^+^ and CD8^+^) cells (Fig. 10). In basal conditions in which thymocytes migrated in the presence of BSA, all DP^hi^ T cell subsets migrated more than those derived from either WT or DP^low^ thymuses, although without significant differences (Fig. 10a). On the contrary, the proportion of migrating DP^low^ thymocytes were significantly lower than WT values in the case of DP and SP CD4^+^ and CD8^+^ thymocytes (Fig. 10a).

Regardless of the origin of the thymocytes analyzed, there were no changes in the proportions of migrating DN thymocytes in any of the experimental conditions tested (Fig. 10). Cell migration was higher in the presence of LN (Fig. 10d) and when LN and CCL21 were combined (Fig. 10e). On the other hand, whereas a significantly higher proportion of DP thymocytes derived from DP^hi^ thymuses migrated in the presence of BSA/CCL21 (Fig. 10b) and BSA/CCL25 (Fig. 10c), those from DP^low^ thymuses showed a lower migratory capacity than WT ones with significant differences in the presence of BSA, as indicated above.

The most important differences occurred in the migration of SP cells, with a significantly smaller proportion of DP^low^ migrating CD4^+^ cells in assays with BSA (Fig. 10a), BSA plus CCL21 (Fig. 10b), BSA plus CCL25 (Fig. 10c), LN (Fig. 10d) and LN and CCL21 (Fig. 10e). In general, the proportion of migratory DP^low^ CD8^+^ cells was also lower with significant differences compared to WT values only in the assays with BSA (Fig. 10a). On the contrary, DP^hi^ CD4^+^ thymocytes migrated significantly more than WT ones in assays with BSA plus CCL25 (Fig. 10c) and LN (Fig. 10d), but significantly less in the presence of LN and CCL21 (Fig. 10e). Finally, the proportions of CD8^+^ cells derived from DP^hi^ thymuses were significantly higher than those of WT ones in the assays established with BSA plus CCL21 or CCL25, and LN plus CCL25 (Fig. 10f).

## Discussion

The present study confirms and extends previous results [8] which demonstrated that the absence of EphA4 affected mutual influences between thymocytes and TECs in the thymus, profoundly altering T cell differentiation, thymic histological organization and the proportions of epithelial cell subsets and various components of the ECM.

The first noticeable result of our study is the existence of mutant mice with different proportions of DP thymocytes, although, really, all the mutants have common features, such as thymic hypocellularity and altered maturation of both thymocytes and thymic epithelium. These apparently contradictory results could merely reflect a temporal delay in the appearance of the severe thymic phenotype of DP^low^ mice, a consequence of the outbred condition of the CD1 mouse strain used. Similar results were reported about the lack of effects of EphA4 on the innervation of dorsal muscles [11] and, in our previous report [8], we remarked that, while at 3 weeks of postnatal life only 42% of mutant mice showed low proportions of DP thymocytes, 4 weeks after birth this proportion increased to 70% of the mutants analyzed.

Nevertheless, it is important to explain the different phenotypes observed in the EphA4-deficient thymuses. In our opinion, the model that best explains the observed T-cell phenotypes of EphA4 mutants containing different proportions of DP cells assumes that the distinct stages of thymocyte maturation respond to the lack of EphA4. Whereas the proportions of DN thymocytes do not change in DP^hi^ thymuses as compared to WT values, in DP^low^ ones they increased significantly. Moreover, in all EphA4-deficient thymuses, the proportions of DN3 cells rose significantly, while reduced proportions of DN4 thymocytes only occurred in EphA4 mutant thymuses containing reduced proportions of DP cells.

Furthermore, within the DN cell compartment, DN3 cells that signal through the so-called preTCR [17] will be selected to progress to the DN4 cell stage and to undergo allelic exclusion and proliferation, whereas non-selected ones will die or differentiate to the γδ T cell lineage [15]. Presumably, in DP^hi^ thymuses a proper β selection of the preTCR courses with decreased proportions of apoptotic cells favoring the proliferation and differentiation of DN3 thymocytes to DN4 and DP cells, thus accounting for the increased proportions of DP cells that occur in these mutants. In the DP^low^ thymuses, the proportion of DN3 accumulates because the proportion of DN4 cells is significantly reduced. Therefore, lower proportions of developing thymocytes reach the DP cell compartment. Hence, despite the increased proportions of cycling DP cells, this shows an increased percentage of apoptotic DP thymocytes, which contributes to the extreme decline in DP thymocytes. Also contributing to this condition would be the reduced proportions of VLA-4^+^ cells observed in the DN cell compartment of DP^low^ thymuses, which, as reported previously [14], could affect their interactions with FN, hence partially impeding the differentiation toward DP thymocytes. In addition, increased proportions of NK cells, DCs and γδ cells reduce the numbers of committed cells to the TCRαβ T cell lineage and, therefore, their presumptive differentiation towards DP cells.

On the other hand, changes in the behavior of DP and SP thymocytes, together with the selective processes involved in generation of the TCR repertoire in the distinct groups of EphA4-deficient mice, could explain the phenotypes found. Thus, in DP^hi^ mutants, reduced proportions of positively selected thymocytes and Treg cells, together with a lack of changes in the proportions of CD69^−^CD62L^+^ cells, would explain the fall in SP thymocytes in these mutants. On the contrary, in both DP^int^ and DP^low^ thymuses, there are increased proportions of positively selected thymocytes and Treg cells without changes in the proportions of negatively selected thymocytes. In these cases, not only does the increased positive selection favor a high differentiation of DP cells to SP thymocytes thus reducing the proportions of the former and increasing the proportions of SP cells, but the accumulation of mature CD69^−^CD62L^+^ CD4^+^/CD8^+^ cells would also contribute to this increase. In this regard, we had suggested previously that a lack of EphA4 would affect both TCRβ and TCRαβ selection [8]. However, to our knowledge there is no evidence for a direct association between EphA4 or their ligands Ephrins A and the thymic selection. In fact, EphA4 expression appears to be regulated by Ktox20/ErgB, a transcription factor involved in thymic selection [18], and the co-stimulation of thymocytes with EphrinA-Fc fusion proteins and anti-CD3 suppress CD69 expression [9]. In addition, EphA4 KO mice show a less severe autoimmune phenotype than WT mice after experimental autoimmune encephalomyelitis (EAE) induction with a MOG peptide [19]. Unfortunately, these authors neither correlate these results with the condition of thymus nor with the proportions of positive and negatively selected thymocytes, concluding that the lack of EphA4 signaling was consistent with a non-inflammatory central nervous system specific deleterious effect of EphA4 [19]. According to our results, if these mice are equivalent to the DP^low^ mutants herein described, the high proportions of Treg cells observed could also explain their decreased autoimmune response***.***

Together with altered T cell differentiation, the distinct EphA4-deficient thymuses show specific thymic epithelial phenotypes. We have repeatedly emphasized a role for Eph and Ephrins in T-TEC interactions [3] and in our previous study on the phenotype of EphA4 KO thymuses, we demonstrated that WT bone marrow lymphoid progenitor cells abnormally differentiated into an alymphoid EphA4-deficient thymic stroma grafted under the renal capsule of SCID mice [8]. On this basis, our current results suggest that the lack of EphA4 impedes the normal T-TEC crosstalk affecting the proper development of both thymocytes and thymic stroma.

The reduced or quasi-normal proportions of DP thymocytes due to the different behavior of DN cells in DP^int^/DP^low^ and DP^hi^ thymuses, respectively, also affect the development of thymic epithelium. In the former, an important absence of DP thymocytes favors increased proportions of both mature and, to a lesser degree, immature Ly51^+^UEA-1^−^ cTECs that obliterate the holes left by the drop in DP thymocytes and eventually collapse of the epithelial cell layers thus reducing the cortical area in these mutant thymuses. Remarkably, in rat thymuses EphA4 is predominantly expressed in the cortex [1]. In these same thymuses, the medullary area increases to house the increased proportions of SP thymocytes resulting from the higher levels of positive selection and Treg cell proportions and the unchanged negative selection and possible retention of CD69^−^CD62L^+^ cells. However, this increase in the medulla area courses with reduced proportions of mTECs due to an impaired maturation of MHCII^lo^UEA-1^+^/CD80^lo^ immature mTECs. Mechanistically, these changes in the cortex and medulla of mutants containing low DP thymocytes, appears to also reflect the role played by EphA4 in cell adhesion.

During development of the nervous system, EphA4 functions as a cell repellent for axon guidance [20]. In addition, the loss of EphA4 is associated with altered dendritic spine morphology [21], and in RTOCs established with DP thymocytes and fetal thymic stromal cells, treatment with EphrinB1-Fc fusion proteins prevents the extension of epithelial cell processes [22]. Furthermore, Sharfe and colleagues [10] pointed out that the absence of EphA4 signaling and activation of Ephrin A mediated by other EphA favor cell attachment. We consider that the reduced proportions of DP thymocytes in the cortex of DP^int/low^ thymuses favor the occupancy of empty intercellular spaces by cTEC that undergo changes in the cell processes, losing their stellate shape to finally flatten, closely pack and collapse. In turn, this thymic cortical organization forces a sponge-like epithelial network of thymic medulla with decreased proportions of mature MHCII^hi^UEA-1^+^/CD80^+^ mTECs.

On the other hand, it is assumed that developing thymocytes move throughout the distinct histological thymic compartments to achieve a proper functional maturation by establishing adequate T-TEC interactions that ensure a final correct T cell maturation. Does the histological organization found in DP^int/low^ thymuses allow this migration of developing thymocytes and/or accurate T-TEC interactions?

In a first approach, we evaluated the migratory capacities of thymocytes derived either from WT, DP^hi^ or DP^low^ thymuses. Remarkably, the migratory capacities of both DP^hi^ and DP^low^ DN cells and DP cells, which occur in the thymic cortex where the most important histological alterations happen, undergo none or only minimal changes, compared to migrating WT T cells. Indeed, CD4^+^ and CD8^+^ SP thymocytes that migrate from cortex to medulla are the T cell subsets whose proportions significantly vary in the distinct experimental conditions, although the proportions of migratory thymocytes were always lower when T cells derived from DP^low^ thymuses were tested. Laminin appears to be the main regulator, among those checked, of migrating CD4^+^ and CD8^+^ thymocytes derived from DP^hi^ thymuses. While WT thymocytes increased their migratory capacity in assays with LN and CCL21, DP^hi^ ones did not; besides, in the presence of LN neither CCL21 nor CCL25 increased the proportions of migratory CD8^+^ cells.

According to current results, the role of chemokines and their receptors does not appear to be key, although the lack of quantitative changes in the expression of these molecules does not necessarily mean that their activation remains unaltered. However, this topic requires further confirmation. The pattern of expression of the assayed chemokines does not change in all EphA4-deficient thymuses, but the proportions of CCR7^+^, the CCL21 receptor; and CCR9^+^, the CCL25 receptor, cells decreased in the DN cells and selected CD4^+^ and CD8^+^CD69^+^ cells of DP^low^ thymuses, supporting low proportions of migrating CD4^+^ cells derived from DP^low^ thymuses in response to CCL21 and CCL25. Further research in experimental conditions directly involving isolated mutant TECs and other combinations of chemokines would confirm these results.

Although in vitro results obtained in transwell assays are difficult to correlate with the phenotypes of EphA4-deficient thymuses observed, we hypothesize that changes in the migratory patterns of the three groups of EphA4 mutants studied do not conclusively support their phenotypes. Instead, altered cell adhesion in both cortical and medullary epithelium would affect T-TEC interactions modifying the thymocyte selection and, in general, T cell differentiation. The increased expression of LN, Col IV and FN in DP^int/low^ thymuses and their interactions with their specific receptors, which also increase in the case of positively selected DP and SP (both CD4^+^ and CD8^+^) cells, could contribute to the adhesion of cTEC in the thymic cortex thus reducing the migration of SP thymocytes. Remarkably, in other experimental models that course with reduced proportions of DP thymocytes there are increased deposits of ECM components [23] that, however, do not occur in DP^hi^ thymuses, in which the cortical epithelium does not collapse.

One final issue deserves a more extensive discussion: the thymic hypocellularity found in the three groups of studied EphA4 mutants that reaches extreme levels in DP^low^ thymuses. In our first study, low cell content was associated with increased proportions of apoptotic DP and SP CD4^+^ cells as well as with reduced cycling DN and DP thymocytes [8]. A similar low cell content is yielded from rat fetal thymic organ cultures (FTOCs) treated with fusion proteins (i.e., EphA1-Fc; EphA2-Fc, EphA3-Fc, Ephrin A1-Fc) [1], and EphA4 has been reported to be involved in neuron survival [24]. In our current study, the low cellularity found in DP^low^ thymuses correlates with low proportions of total cycling cells, but the percentage of cycling DP thymocytes significantly increased. In addition, changes in the proportions of apoptotic cells could not explain the extreme hypocellularity of DP^low^ thymuses. Moreover, although the proportions of apoptotic DP^low^ cells were high, the cycling cells decreased in the DN cell subset respect to WT values. On the other hand, in the case of DP^hi^ thymuses, it is more difficult to establish the origin of thymic hypocellularity because the proportion of both apoptotic DN and DP thymocytes significantly decreases and that of the cycling cells does not change.

It is important to remark that, as previously reported [8], our results evaluated at three weeks of postnatal life reflect cumulative alterations in the survival/death, proliferation and differentiation of mutant thymic cells that begin at the end of fetal life [8]. Undoubtedly, thymic macrophages engulf apoptotic cells, and, at the same time, proliferating cell progenitors try to balance this cell decline, masking the results corresponding to any fixed moment in time.

## Supplementary Information

Below is the link to the electronic supplementary material.

Supplementary file1 (DOCX 1869 KB)

## Data Availability

The data generated during the study are available from the corresponding author upon reasonable request.
