# Supplementary material for: Altered thymocyte development observed in EphA4-deficient mice courses with changes in both thymic epithelial and extracellular matrix organization
Source: Cell Mol Life Sci. 2022 Nov 5;79(11):583. doi: 10.1007/s00018-022-04610-w (PMC9637064; doi:10.1007/s00018-022-04610-w)
Supplement: Supplementary file 1 — Supplementary file1 (DOCX 1869 KB) [file 18_2022_4610_MOESM1_ESM.docx]

**Altered thymocyte development observed in EphA4-deficient mice courses with changes in both thymic epithelial and extracellular matrix organization**

Javier García-Ceca^1,2^*, Sara Montero-Herradón^1,2^*, Ana González^1^, Rosa Plaza^1^ and Agustín G Zapata^1,2^†

^1^ Department of Cell Biology, Faculty of Biology, Complutense University of Madrid, 28040 Madrid, Spain.

^2^ Health Research Institute, Hospital 12 de Octubre (imas12), 28041 Madrid, Spain.

* These authors contribute equally to the study

† Corresponding author: Agustín G Zapata; Department of Cell Biology, Faculty of Biology, Complutense University of Madrid, 28040 Madrid, Spain. zapata@ucm.es

ORCID: García-Ceca, J: 0000-0002-0940-455X; Montero-Herradón S: 0000-0003-2004-8987; Zapata AG: 0000-0003-0576-2672

| **Antibody** | **Fluorochrome** | **Clone** | **Company** |
| --- | --- | --- | --- |
| anti-CD4 | APC, FITC | GK1.5 | Biolegend |
| anti-CD8α | APC, PE, Pacific Blue | 53-6.7 | Biolegend |
| anti-TCRβ | FITC | H57-597 | Biolegend |
| anti-CD44 | FITC | IM7 | Biolegend |
| anti-cKit (CD117) | PE | 2B8 | Biolegend |
| anti-CD5 | FITC | 53-7.3 | Biolegend |
| anti-CD69 | APC | H1.2F3 | Biolegend |
| Anti-lineage (Lin) cocktail | APC | --- | BD Biosciences |
| anti-CD25 | PerCP | PC61 | Biolegend |
| anti-CD45 | PerCP, APCCy7, Pacific Blue | 30-F11 | Biolegend |
| anti-EpCAM (CD326) | AlexaFluor488, PE | G8.8 | Biolegend |
| anti-Ly51 (CD249) | PE, AlexaFluor647 | 6C3 | Biolegend |
| anti-MHCII (IA/IE) | PerCP | M5/114.15.2 | Biolegend |
| anti-CD80 | APC | 16-10A1 | Biolegend |
| UEA1-Biotin | --- | --- | Vector Labs |
| anti-TCRγδ | PE | GL3 | Biolegend |
| anti-CD11c | PE | N418 | Biolegend |
| anti-CD49b | PE | DX5 | Biolegend |
| anti-F4/80 | PE | BM8 | Biolegend |
| anti-CD62L | PerCP | MEL-14 | Biolegend |
| anti-cleaved Caspase-3 | PE | polyclonal | Cell Signalling |
| anti-FoxP3 | PE | FJK-16s | eBioscience |
| anti-VLA4 (CD49d) | PE | R1-2 | Biolegend |
| anti-VLA6 (CD49f) | PE | GoH3 | Biolegend |
| anti-CXCR4 | PE | 2B11 | eBioscience |
| anti-CCR9 | PE | eBioCW-1.2 | eBioscience |
| anti-CCR7 | PE | 4B12 | eBioscience |
| anti-RatIgG2b,k | PE | RTK4530 | Biolegend |
| anti-MouseIgG2a,k | PE | MOPC-173 | Biolegend |
| anti-RatIgG2a,k | PE | RTK2758 | Biolegend |
| anti-Armenian HamsterIgG | PE | HTK888 | Biolegend |
| AnnexinV | BrilliantViolet605, APC | --- | Biolegend |
| Streptavidin | PECy7/PerCP | --- | Biolegend |

**Online Resource 1.** Flow cytometry antibodies

| **Antibody** | **Fluorochrome** | **Clone** | **Company** |
| --- | --- | --- | --- |
| anti-keratin 8 (K8) | --- | Troma-1 | Developmental Studies Hybridoma Bank |
| anti-keratin 5 (K5) | --- | AF138 | Covance |
| anti-mouse thymic stroma 10 (MTS10) | --- | --- | kindly gifted by Ann Chidgey, Monash University |
| anti-Pan Cytokeratin | FITC | C11 | Sigma |
| anti-laminin | --- | polyclonal | Sigma |
| anti-fibronectin | --- | polyclonal | Novotec |
| anti-collagen type IV | --- | polyclonal | Novotec |
| anti-CCL21 | --- | polyclonal | R&D Systems |
| anti-CCL25 | --- | polyclonal | R&D Systems |
| anti-CXCL12 | --- | polyclonal | Fitzgerald |
| anti-AIRE | AlexaFluor488 | 5H12 | eBioscience |
| Goat anti-Rat IgMDyLight | AlexaFluor594 | --- | ThermoFisher Scientific |
| Donkey anti-Rat IgG | AlexaFluor594, 488 | --- | ThermoFisher Scientific |
| Donkey anti-Goat IgG | AlexaFluor488 | --- | ThermoFisher Scientific |
| Donkey anti-Rabbit IgG | AMCA | --- | Jackson InmunoResearch |
| Donkey anti-Rabbit IgG | AlexaFluor488 | --- | ThermoFisher Scientific |

**Online Resource 2.** Immunofluorescence antibodies

**
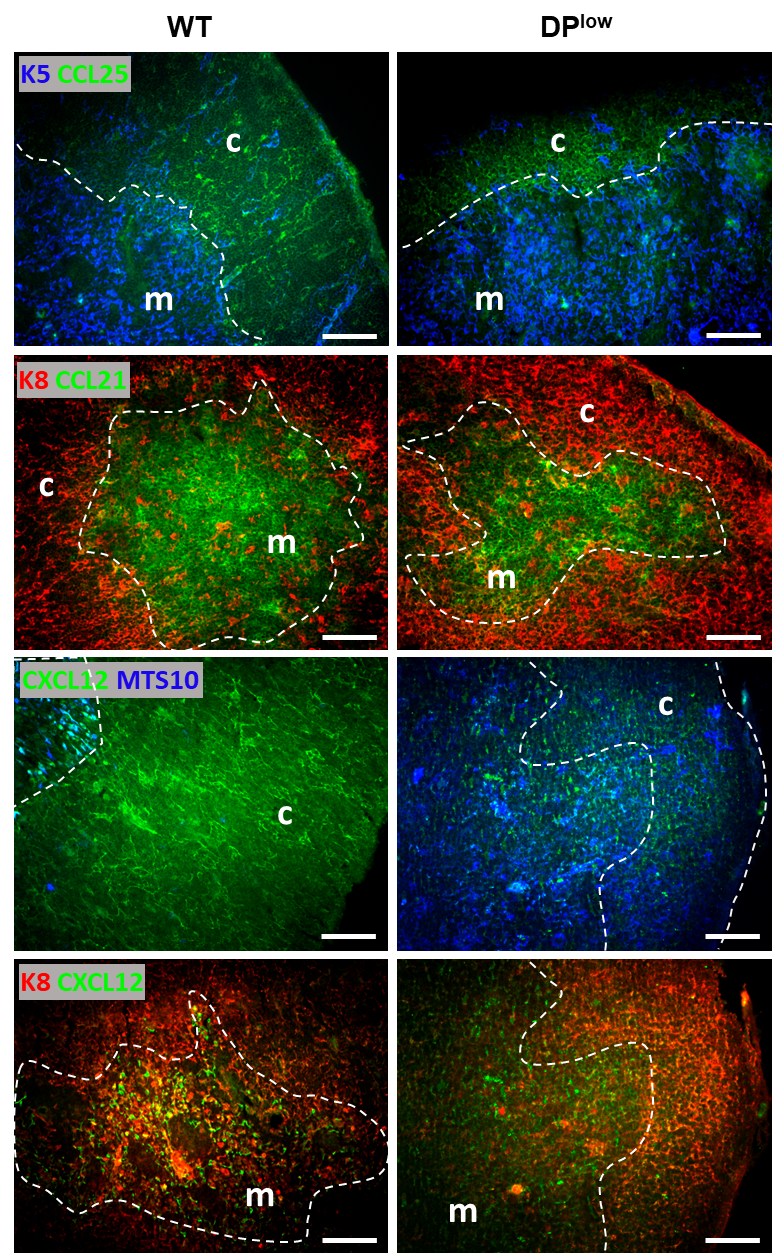
**

**Online Resource 3.** Expression of CCL25, CCL21 and CXCL12 (green) in the thymic cortex (c) stained with keratin 8 (K8, red) or in medulla (m) stained with MTS10^+^ or keratin 5^+^ (K5) (blue) in both WT and DP^low^ EphA4 mutant thymuses. Scale bar: 100µm
